# Novel therapies improve prognosis of IgAN and limit the applicability of the International IgA Nephropathy Prediction Tool

**DOI:** 10.1093/ckj/sfaf251

**Published:** 2025-08-07

**Authors:** Xue Shen, Pei Chen, Lijun Liu, Sufang Shi, Xujie Zhou, Sean J Barbour, Jicheng Lv, Hong Zhang

**Affiliations:** Renal Division, Department of Medicine, Peking University First Hospital, Beijing, China; Institute of Nephrology, Peking University, Beijing, China; Key Laboratory of Renal Disease, Ministry of Health of China, Beijing, China; Key Laboratory of Chronic Kidney Disease Prevention and Treatment (Peking University), Ministry of Education, Beijing, China; Renal Division, Department of Medicine, Peking University First Hospital, Beijing, China; Institute of Nephrology, Peking University, Beijing, China; Key Laboratory of Renal Disease, Ministry of Health of China, Beijing, China; Key Laboratory of Chronic Kidney Disease Prevention and Treatment (Peking University), Ministry of Education, Beijing, China; Renal Division, Department of Medicine, Peking University First Hospital, Beijing, China; Institute of Nephrology, Peking University, Beijing, China; Key Laboratory of Renal Disease, Ministry of Health of China, Beijing, China; Key Laboratory of Chronic Kidney Disease Prevention and Treatment (Peking University), Ministry of Education, Beijing, China; Renal Division, Department of Medicine, Peking University First Hospital, Beijing, China; Institute of Nephrology, Peking University, Beijing, China; Key Laboratory of Renal Disease, Ministry of Health of China, Beijing, China; Key Laboratory of Chronic Kidney Disease Prevention and Treatment (Peking University), Ministry of Education, Beijing, China; Renal Division, Department of Medicine, Peking University First Hospital, Beijing, China; Institute of Nephrology, Peking University, Beijing, China; Key Laboratory of Renal Disease, Ministry of Health of China, Beijing, China; Key Laboratory of Chronic Kidney Disease Prevention and Treatment (Peking University), Ministry of Education, Beijing, China; BC Renal, Provincial Health Services Authority, Vancouver, British Columbia, Canada; Division of Nephrology, Department of Medicine, University of British Columbia, Vancouver, British Columbia, Canada; Renal Division, Department of Medicine, Peking University First Hospital, Beijing, China; Institute of Nephrology, Peking University, Beijing, China; Key Laboratory of Renal Disease, Ministry of Health of China, Beijing, China; Key Laboratory of Chronic Kidney Disease Prevention and Treatment (Peking University), Ministry of Education, Beijing, China; Renal Division, Department of Medicine, Peking University First Hospital, Beijing, China; Institute of Nephrology, Peking University, Beijing, China; Key Laboratory of Renal Disease, Ministry of Health of China, Beijing, China; Key Laboratory of Chronic Kidney Disease Prevention and Treatment (Peking University), Ministry of Education, Beijing, China

**Keywords:** disease progression, end-stage kidney disease, IgA nephropathy, novel therapies, risk prediction tool

## Abstract

**Background:**

The International IgA Nephropathy Prediction Tools using clinical variables and the Oxford MEST scores were developed in outdated cohorts. External validation is required to assess the tool's applicability in predicting progression risk for patients on novel therapies.

**Methods:**

We included 677 immunoglobulin A nephropathy (IgAN) patients (Peking University First Hospital, 2003–23) treated with endothelin receptor antagonists, Nefecon, sodium-glucose cotransporter 2 inhibitors, hydroxychloroquine or telitacicept, a BAFF/APRIL inhibitor. The primary outcome was defined as a 50% decline in estimated glomerular filtration rate or end-stage kidney disease. Discrimination (C-statistic), calibration [calibration slope, Integrated Calibration Index (ICI)], model fit (R^2^_D_) and risk stratification (Kaplan–Meier curves) were assessed.

**Results:**

The median follow-up was 4.8 years (interquartile range 2.2, 8.1), and 190 (28.1%) patients experienced the primary outcome, with a 5-year risk of 9.8%. Compared with the median biopsy year of reported cohorts of original model, our cohort is more contemporary (2017). We validated both original and updated models (and for full model with and without race version). All versions showed adequate discrimination, poor calibration and model fit: C-statistic ∼0.74, calibration slope ∼0.50, R^2^_D_ <20%, ICI >0.10, and poor separation of Kaplan–Meier curves, except for the highest-risk group. The tools consistently overestimated risk in patients receiving novel therapies. These findings further demonstrated that novel therapies can improved clinical outcomes for IgAN patients.

**Conclusions:**

In this study, both versions of both models demonstrated limited performance and overestimated risks. Given the prognostic improvement with novel IgAN therapies, these prediction tools may need updating for use in currently treated patients.

## Graphical Abstract

**Graphical Abstract:**
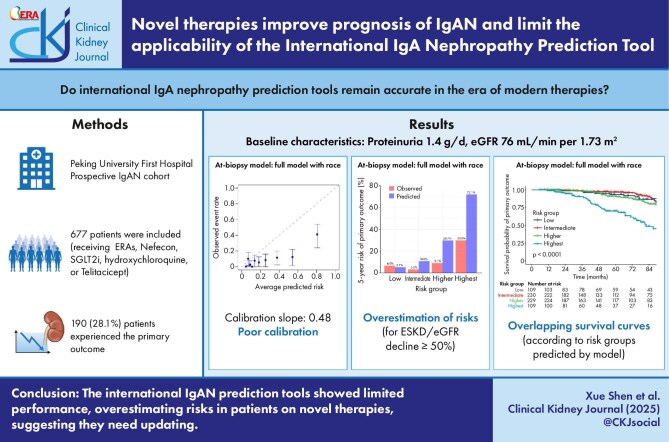

KEY LEARNING POINTS **What was known:**

- The International IgA Nephropathy Prediction Tools performed well in earlier validation studies of historical cohorts.
- However, their applicability remained untested in patients receiving new supportive and disease-modifying therapies like Nefecon, sodium-glucose cotransporter 2 inhibitors and endothelin receptor antagonists.

**This study adds:**

- This study demonstrates that these tools, when applied to a contemporary cohort treated with novel therapies, exhibit poor calibration and consistent risk overestimation, highlighting their limited utility in current practice.

**Potential impact:**

- The results underscore the need to update or refine existing prediction models to align with modern treatment approaches, ensuring more accurate risk stratification for improved patient management.
- From an alternative perspective, these findings suggest that the novel therapies can improve clinical outcomes.

## INTRODUCTION

Immunoglobin A nephropathy (IgAN), the most common primary glomerulonephritis globally, is a leading cause of chronic kidney disease and end-stage kidney disease (ESKD) [1].

While earlier studies suggested 20%–40% of patients progress to ESKD within 20 years post-diagnosis [1–3], recent large-scale cohort studies reveal more severe outcomes. A study conducted in the UK reported a 10-year kidney survival rate of only 54% [4], consistent with our Chinese cohort finding of 61% [5], indicating that most IgAN patients progress to ESKD within 10–15 years. These findings highlighted the poor long-term outcomes of IgAN, underscoring the importance for early diagnosis and precise outcome prediction to guide treatment decisions.

The Kidney Disease: Improving Global Outcomes (KDIGO) 2024 Clinical Practice Guidelines (draft) [6] recommended using clinical and pathological data at kidney biopsy for risk stratification in IgAN, identifying the International IgAN Prediction Tool (IIgAN-PT) as a valuable tool for assessing progression risk. Developed by Barbour *et al*. in 2019, the IIgAN-PT included variables such as estimated glomerular filtration rate (eGFR), blood pressure, proteinuria, MEST histologic scores, age, racial/ethnic characteristics and medication use [including renin–angiotensin system (RAS) blockade and immunosuppressants]. This tool predicted the risk of primary outcome within 80 months in adults based on clinicopathological data at biopsy [7]. Subsequent updates in 2020, 2022 and 2024 expanded its application to pediatric patients at biopsy and both adult and pediatric patients 1–2 years post-biopsy [8–10]. Beyond internal validation, external validation studies, primarily conducted in China and South Korea, had consistently validated the performance of the IIgAN-PT, particularly the 2019 adult model [11–15].

The treatment regimens for IgAN evolved beyond conventional RAS blockers (RASB) and immunosuppressive therapies, with over 20 novel therapies in clinical development. These therapies targeted diverse mechanisms, including B cell/plasma cell depletion (e.g. CD20 and CD38 monoclonal antibodies), B cell/plasma cell modulation (e.g. Nefecon, BAFF/APRIL inhibitors), targeting immune complex–mediated glomerular inflammation (e.g. C3 and C5 inhibitors) and managing nephron loss [e.g. sodium-glucose cotransporter-2 inhibitors (SGLT2i), endothelin receptor antagonists] [16]. Multiple phase 2–3 clinical trials demonstrated their potential to significantly improve patient outcomes [17–22]. Among these drugs, endothelin receptor antagonists (ERAs), Nefecon, SGLT2i, hydroxychloroquine and telitacicept have been widely used in clinical practice.

Although the IIgAN-PT was not intended to guide therapy or assess treatment efficacy, its relevance for outcome risk prediction in patients receiving novel therapies remains unclear, as the derivation cohort used to develop the tool had a median biopsy year of 2006 [7], a period when treatment approaches were markedly distinct from current practices.

In this study, we performed comprehensive external validation of the IIgAN-PT in patients using new drugs including ERAs, Nefecon, SGLT2i, hydroxychloroquine and telitacicept. External validation was conducted for two models: original model and updated model for adults [7, 9]. Both versions of the full model with and without race were validated. Additionally, we input new drugs as prior variables RASB and immunosuppressant. Our findings will inform the potential role of IIgAN-PT in predicting patient outcomes with receiving new therapies and highlight the potential need for model updates.

## MATERIALS AND METHODS

### Study participants

An observational, prospective cohort study was conducted at Peking University First Hospital from January 2003 to June 2023. We included patients with biopsy-proven primary IgAN and follow-up data of ≥1 year. Patients with secondary IgAN due to systemic diseases, IgAN in transplanted kidneys, conditions affecting treatment or ESKD at the time of biopsy were excluded. A total of 2268 IgAN patients were included in our cohort. We further selected patients who received novel treatments, including ERAs, Nefecon, SGLT2i, hydroxychloroquine and telitacicept, a BAFF/APRIL inhibitor, requiring a minimum of 12 months of eGFR data post-initiation of the novel treatment.

The study was approved by the Clinical Research Ethics Committee of Peking University First Hospital [Ethics Approval Number: 2013 (548)] and was conducted in accordance with the principles of the Declaration of Helsinki. All patients gave fully informed written consent. The ethnicity of all participants was Chinese.

### Predictors and outcome

For the IIgAN-PT published in 2019 (referred to as the “At-biopsy model”), baseline characteristics, including proteinuria, mean arterial pressure (MAP), eGFR (calculated using the Chronic Kidney Disease Epidemiology Collaboration formula [23]), age, and prior use of RASBs and immunosuppressants, were collected at the time of biopsy. Oxford classification MEST scores were assessed by two experienced pathologists. The full model with race incorporated additional race information (Chinese, Japanese, White or other). For the IIgAN-PT published in 2022 (referred to as “Post-biopsy model”), which predicts outcome risks based on data collected 1–2 years after kidney biopsy, the predictive factors include the above-mentioned clinicopathological characteristics at the landmark time after biopsy, with RASB or immunosuppressant use at or prior to the landmark time post-biopsy.

For both the At-biopsy model and Post-biopsy model, the primary outcome was defined as a composite of the first occurrence of either ESKD (eGFR <15 mL/min/1.73 m², dialysis or kidney transplantation) or a 50% decline in eGFR.

### Prediction models for external validation

We conducted four external validations: (i) external validation of the At-biopsy model in the new drug cohort; (ii) external validation of the At-biopsy model using exclusively ERA- or SGLT2i-treated patients in the new drug cohort; (iii) external validation of the Post-biopsy model using characteristics at 1 year post-biopsy in the new drug cohort; (iv) external validation of the Post-biopsy model by inputting the use of ERAs and SGLT2i as RASBs, and the use of Nefecon, hydroxychloroquine and telitacicept as immunosuppressants.

Following the formula provided by Barbour *et al*. [7, 9], we calculated the linear predictor and the prediction probability of the primary outcome for each patient. The specific calculation formulas are shown in Supplementary data, Table S1. In the full model with race, a piecewise function was applied to the Chinese race at 36 months due to a violation of the proportional hazards assumption. Using the same method as the original study, we classified patients into four risk groups based on the centiles of the linear predictors: low risk (<16th percentile), intermediate risk (16th–50th percentile), higher risk (50th–84th percentile) and highest risk (>84th percentile).

We followed the TRIPOD (Transparent Reporting of a multivariable prediction model for Individual Prognosis Or Diagnosis) reporting guidelines for this study (Supplementary data, Table Appendix, TRIPOD Checklist).

### Statistics analysis

We estimated the minimum required sample size for each prognostic model using the pmsampsize R package (Supplementary data, Table S2) [24].

We evaluate model performance from three aspects. For discrimination and model fit, the C-statistic, calibration slope and R²_D_ were calculated. The C-statistic, namely the area under the receiver operating characteristic curve, was computed according to Chambless *et al*. to assess discrimination [25], and evaluated using the 5-year risk of the composite outcome (as this corresponded to the median follow-up time of the cohort). The calibration slope, the coefficient of the linear predictor, was then calculated by fitting a Cox model to regress the primary outcome with the linear predictor as the only variable. A slope value >1 indicates greater discrimination. Model fit was further assessed using the coefficient of determination (R²_D_), where an increase in R²_D_ indicated a better model fit [26].

For calibration, the predicted and observed risks for the primary outcome were compared. We calculated the Integrated Calibration Index (ICI) based on the 5-year risk. The ICI is the weighted difference between the observed proportions and the predicted probabilities [27]. A calibration plot was created by dividing patients into 10 groups based on the deciles of the linear predictors. The average 5-year predicted risk per group was compared with the observed Kaplan–Meier risk. Bar charts further compared predicted and observed risks across low-, intermediate-, high- and highest-risk groups.

We compared the four risk groups using Kaplan–Meier survival curves for the primary outcome. eGFR slopes were derived from a linear mixed-effects model (R “lme4”), adjusted for age, sex, MAP, 24-h urinary protein excretion rate (24-h UP) and MEST scores.

Statistical significance was defined as a *P*-value <.05, with two-tailed tests. Statistical analyses were conducted using R Version 4.4.2 (R Foundation for Statistical Computing, Vienna, Austria).

## RESULTS

### Study population characteristics

**Figure 1:**
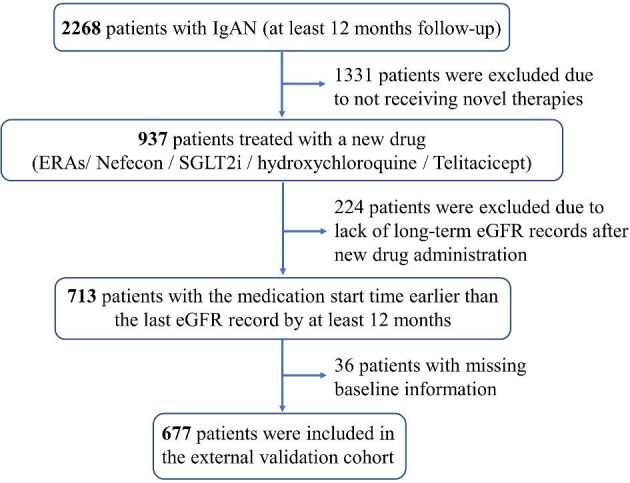
Study participant inclusion and exclusion flowchart. According to the inclusion and exclusion criteria, 677 IgAN patients received novel drugs were finally enrolled in the study.

**Figure 2:**
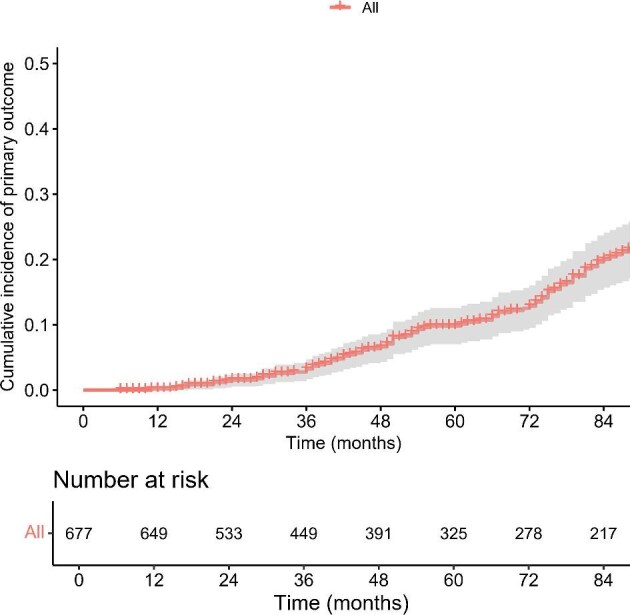
The Kaplan–Meier survival curves of the included patients. A total of 677 IgAN patients received novel drugs were enrolled in our study, and the 5-year incidence of the primary endpoint was 9.8%.

The inclusion and exclusion flowchart for the new drug cohort is shown in Fig. 1, which included a total of 677 patients. Over a median 4.8 years of follow-up after biopsy [interquartile range (IQR) 2.2, 8.1], 190 (28.1%) patients experienced the primary outcome (eGFR decline ≥50% or ESKD), with a 5-year primary outcome risk of 9.8% (Fig. 2). The primary outcome rate was higher than the reported derivation and validation cohorts (17.7% and 18.6%, respectively) [7]. The follow-up period was similar to that of the reported derivation and validation cohorts (4.8 and 5.8 years, respectively) [7]. Detailed medication regimens are presented in Supplementary data, Table S3, with the four most common treatment combinations all including hydroxychloroquine. Eighty-two patients were treated with new drugs that only included ERAs, SGLT2i or their combination; 10 of these patients experienced the primary outcome.

The baseline characteristics of our external validation cohort compared with the reported cohorts are shown in Table 1. Compared with the reported cohorts (2006 and 1998, respectively) [7], our external validation cohort is more contemporary (2017), as it only includes patients treated with new drugs. The application rates of RASB (33.7% versus 32.4% and 32.0%, respectively) and immunosuppressant (7.8% versus 9.1% and 7.1%, respectively) at or prior to kidney biopsy in the external validation cohort were similar to those in the reported cohorts. The median age at kidney biopsy in our external validation cohort was 22 years, with slightly lower baseline eGFR and slightly higher baseline proteinuria than those in the reported cohorts. The proportions of M1, E1 and T1/2 scores and crescents in the Oxford MEST-C histologic scores were also higher in our external validation cohort, indicating a more severe disease state in our external validation cohort.

**Table 1:** Characteristics of participants in the reported cohorts and the external validation cohort at the time of kidney biopsy.

Characteristics	Reported derivation cohort	Reported validation cohort	Our validation cohort
Number of patients	2781	1146	677
Follow-up time, median (IQR), years	4.8 (3.0–7.6)	5.8 (3.4–8.5)	4.8 (2.2–8.1)
Year of biopsy, median (IQR)	2006 (2004–2008)	1998 (1993–2003)	2017 (2014–2020)
Age, median (IQR), years	35.6 (28.2–45.4)	34.8 (26.9–45.0)	22.0 (17.0–29.0)
Male, *n* (%)	1608 (57.8)	565 (49.3)	313 (46.2)
Race, *n* (%)			
Caucasian	1167 (42.0)	176 (15.5)	0 (0)
Chinese	1021 (36.7)	292 (25.8)	677 (100.0)
Japanese	569 (20.5)	616 (54.4)	0 (0)
Other	22 (0.8)	49 (4.3)	0 (0)
Scr at biopsy, median (IQR), μmol/L	92.0 (70.7–123.8)	84.0 (66.2–111.4)	97.0 (77.0–130.0)
eGFR at biopsy, median (IQR), mL/min/1.73 m^2^	83.0 (56.7–108.0)	89.7 (65.3–112.7)	76.0 (51.0–98.0)
<30, *n* (%)	142 (5.1)	37 (3.2)	35 (5.2)
∼30–60, *n* (%)	657 (23.6)	191 (16.7)	193 (28.5)
∼60–90, *n* (%)	800 (28.8)	350 (30.5)	224 (33.1)
≥90, *n* (%)	1182 (42.5)	568 (49.6)	225 (33.2)
MAP at biopsy, median (IQR), mmHg	96.7 (88.7–106.3)	93.3 (85.0–103.3)	99.0 (90.0–108.0)
Proteinuria at biopsy, median (IQR), g/day	1.2 (0.7–2.2)	1.3 (0.6–2.4)	1.4 (0.8–2.5)
<0.5, *n* (%)	383 (13.9)	221 (19.4)	70 (10.3)
∼0.5–1, *n* (%)	772 (28.1)	209 (18.3)	151 (22.3)
∼1–2, *n* (%)	817 (29.7)	352 (30.8)	220 (32.5)
∼2–3, *n* (%)	360 (13.1)	145 (12.7)	106 (15.7)
≥3, *n* (%)	415 (15.1)	215 (18.8)	130 (19.2)
MEST histologic score, *n* (%)			
M1	1054 (38.0)	481 (42.0)	344 (50.8)
E1	478 (17.3)	476 (41.5)	250 (36.9)
S1	2137 (77.0)	912 (79.6)	514 (75.9)
T1	686 (24.7)	207 (18.1)	179 (26.4)
T2	128 (4.6)	122 (10.6)	59 (8.7)
Crescents	953 (34.3)	642 (56.1)	461/666 (69.2)^a^
RASB use before or at biopsy, *n* (%)	862 (32.4)	320 (30.0)	228 (33.7)
Immunosuppressant use before or at biopsy, *n* (%)	252 (9.1)	81 (7.1)	53 (7.8)
Primary outcome, *n* (%)			
50% decline in eGFR	420 (15.1)	210 (18.3)	187 (27.6)
ESKD	372 (13.4)	155 (13.5)	132 (19.5)
Total primary outcomes	492 (17.7)	213 (18.6)	190 (28.1)

Data are presented median (Q1, Q3), mean ± standard deviation or *n* (%).

The data of reported cohorts refer to the data published by Barbour *et al*. in 2019 [7].

^a^Data are shown as *n*/*n* (%) because of incomplete crescent data.

In the new drug cohort, the median time from kidney biopsy to receiving new drug was 1.4 years (IQR 0.3, 4.4). We conducted an external validation of the Post-biopsy model, a total of 566 patients with complete data at 1 year post-biopsy were included, and the clinicopathological information at 1 year post-biopsy is presented in Supplementary data, Table S4.

### Performance of the international IgA nephropathy prediction tool

#### Discrimination and model fit

Table 2 presents the discrimination and model fit measures. In our new drug cohort, the At-biopsy model showed C-statistics of 0.740 (with race version) and 0.735 (without race version), lower than the reported cohort's 0.82 and 0.81. Notably, patients receiving exclusively ERAs or SGLT2i achieved superior C-statistics (0.850 and 0.885, respectively). The Post-biopsy model similarly demonstrated 0.736/0.733 (vs 0.87/0.86 in reported cohort). Reclassifying new drugs as RASB/immunosuppressants remained suboptimal (0.749/0.752). Calibration slope values for all models and versions across four validations were consistently <1. For model fit, our external validation results showed that the R²_D_ was around 20% or below. This is much lower than ∼25% in the reported cohorts for the At-biopsy model, and ∼60% in the reported cohorts for the Post-biopsy model.

**Table 2:** Discrimination, calibration and model fit measures in our external validation cohorts.

Prediction tool	At-biopsy model		At-biopsy model		Post-biopsy model		Post-biopsy model	
Participants	All patients from the new drug cohort (*N* = 677)		Patients received ERAs and SGLT2i (*N* = 82)		All patients from the new drug cohort (*N* = 566)		All patients from the new drug cohort and putting new drugs under the prior variables (*N* = 566)	
Variable	With race	Without race	With race	Without race	With race	Without race	With race	Without race
C-statistic	0.740	0.735	0.850	0.885	0.736	0.733	0.749	0.752
Calibration slope	0.48	0.49	0.64	0.78	0.47	0.50	0.47	0.50
ICI	0.18	0.12	0.19	0.12	0.14	0.13	0.13	0.12
R²_D_, %	17.6	17.8	16.5	17.6	19.6	20.2	18.4	19.6

Overall, both the At-biopsy and Post-biopsy models exhibited adequate discrimination but poor model fit when applied to the new drug cohort and in subsequent analyses.

#### Model calibration

**Figure 3:**
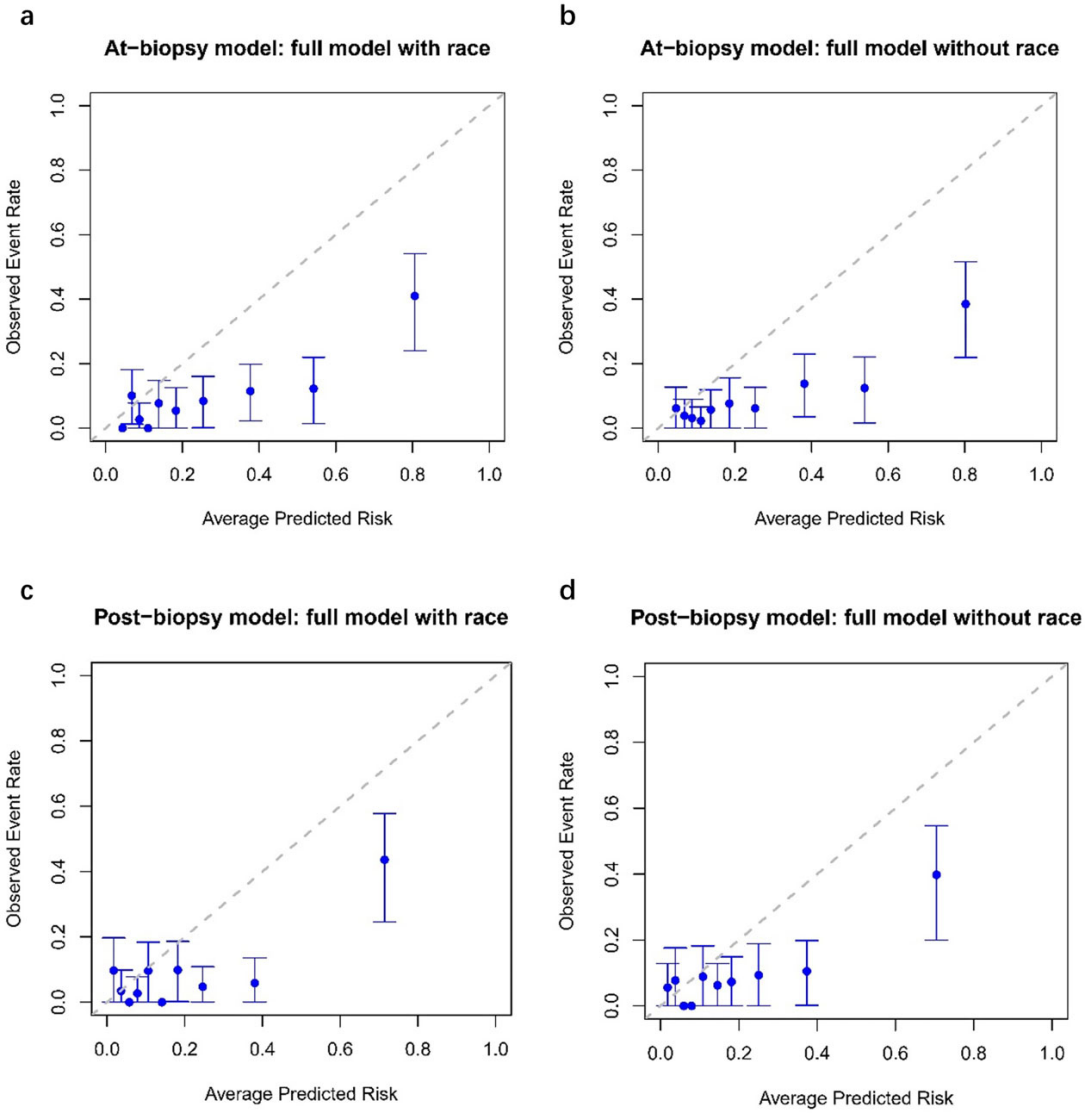
Calibration plots demonstrated the At-biopsy model and Post-biopsy model generally overestimated the risk of primary outcome. (**a, b**) Full model with and without race version of the At-biopsy model. (**c, d**) Full model with and without race version of the Post-biopsy model. Patients were divided into 10 groups according to the deciles of predicted risk derived from each version of each model. The 5-year observed risk and predicted risk were compared among groups. The dashed line represents perfect calibration, namely the predicted risk is exactly the same as the observed risk. Vertical lines parallel to the vertical axis represent the 95% confidence interval of the observed risk for each group.

**Figure 4:**
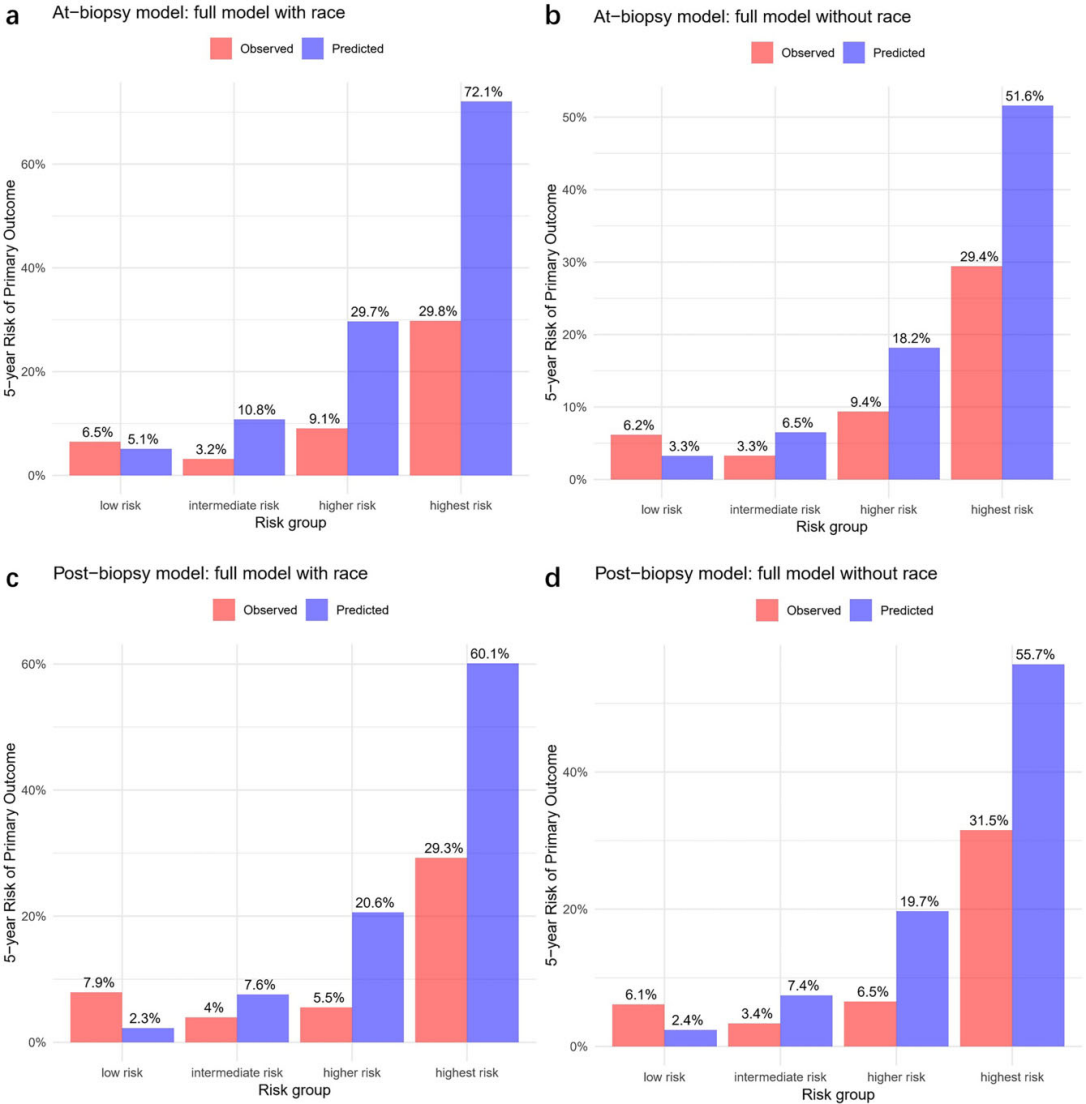
Plots according to risk groups showed the differences between 5-year observed risk and predicted risk in (**a, b**) full model with and without race version of the At-biopsy model and (**c, d**) full model with and without race version of the Post-biopsy model. Risk groups were on the basis of percentiles of the linear predictor (low risk: <16th; intermediate risk: 16th–50th; higher risk: 50th–84th; and highest risk: >84th).

As shown in Table 2, the ICI values for multiple external validations were all >0.1, indicating poor calibration performance. The observed and predicted 5-year risks based on tenths of predicted risk and risk subgroups were shown in Figs 3 and 4. Calibration plots (Fig. 3) revealed apparent differences between observed and predicted risks at 5 years. Both At-biopsy and Post-biopsy models overestimated the primary outcome risk in intermediate-, higher- and highest-risk groups, regardless of race inclusion (Fig. 4). Supplementary data, Figs S1 and S2 confirmed this overestimation pattern through model revalidation: the At-biopsy model was retested in patients receiving ERAs or SGLT2i therapies, while the Post-biopsy model was retested in putting new drugs under prior variables.

#### Comparison of risk groups

**Figure 5:**
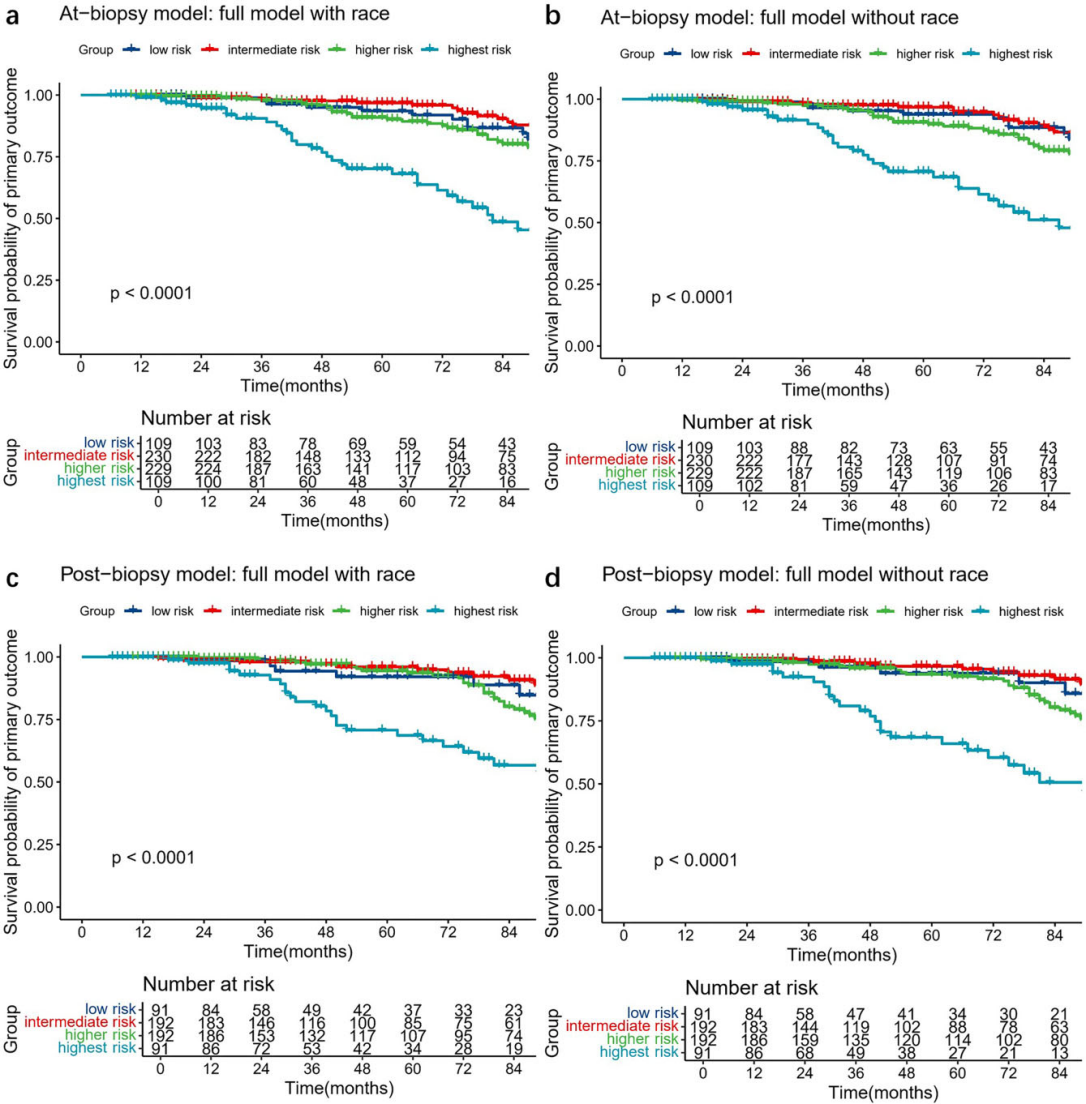
Kaplan–Meier curves of the primary outcome between the risk groups showed poor distinguishing ability in intermediate risk, higher risk and highest risk groups in (**a, b**) full model with and without race version of the At-biopsy model and (**c, d**) full model with and without race version of the Post-biopsy model. Risk groups were on the basis of percentiles of the linear predictor (low risk: <16th; intermediate risk: 16th–50th; higher risk: 50th–84th; and highest risk: >84th).

Our external validation analyses revealed that in both the At-biopsy and Post-biopsy models, only the highest-risk group demonstrated clear curve separation, regardless of race inclusion (Fig. 5). Low-, intermediate- and higher-risk groups exhibited overlapping survival curves across models. Similar patterns emerged in revalidation of models: At-biopsy model in ERA/SGLT2i patients, Post-biopsy model with new drugs as prior variables (Supplementary data, Fig. S3). On the whole, models had difficulty in distinguishing between risk groups other than the highest risk.

The hazard ratios and eGFR slopes for different risk subgroups are shown in Tables 3 and 4. External Validation of the At-biopsy model (Table 3) revealed no significant differences in eGFR slopes among the four risk subgroups, the eGFR slopes in the four subgroups were –2.94, –2.67, –2.55 and –2.64 for the full model with race, and –2.88, –2.62, –2.55 and –2.72 mL/min/1.73 m^2^/year for the full model without race. The hazard ratios between risk groups were well-maintained in the higher and highest-risk group. For the Post-biopsy model (Table 4), the more high-risk subgroup had a slower decline in eGFR, with hazard ratios well-maintained only in the highest-risk group. These results indicated that the risk groups did not align well with the hazard ratios or eGFR slopes.

**Table 3:** Hazard ratios and rate of kidney function decline in subgroups based on linear predictor from the external validation of the At-biopsy model.

Risk group	Number of patients	Events, *n* (%)	HR (95% CI)	*P*-value	Rate of eGFR decline (95% CI), mL/min/1.73 m^2^/year
Full model without race					
Low risk	109	28 (26)	Reference		–2.94 (–3.04 to –2.84)
Intermediate risk	230	52 (23)	1.03 (0.65 to 1.64)	.9	–2.67 (–2.90 to –2.44)
Higher risk	229	72 (31)	1.59 (1.02 to 2.48)	.04	–2.55 (–2.77 to –2.32)
Highest risk	109	38 (36)	4.23 (2.55 to 7.02)	<.001	–2.64 (–2.92 to –2.35)
* P*-value for trend				<.001	
Full model with race					
Low risk	109	26 (24)	Reference		–2.88 (–2.98 to –2.77)
Intermediate risk	230	55 (24)	1.05 (0.96 to 1.68)	.85	–2.62 (–2.85 to –2.38)
Higher risk	229	68 (30)	1.57 (0.99 to 2.49)	.06	–2.55 (–2.78 to –2.32)
Highest risk	109	41 (38)	5.71 (3.40 to 9.61)	<.001	–2.72 (–3.34 to –2.73)
*P*-value for trend				<.001	

HR: hazard ratio; CI: confidence interval.

**Table 4:** Hazard ratios and rate of kidney function decline in subgroups based on linear predictor from the external validation of the Post-biopsy model.

Risk group	Number of patients	Events, *n* (%)	HR (95% CI)	*P*-value	Rate of eGFR decline (95% CI), mL/min/1.73 m^2^/year
Full model without race					
Low risk	91	12 (13)	Reference		–3.04 (–3.17 to –2.90)
Intermediate risk	192	36 (19)	1.10 (0.57 to 2.13)	.77	–2.60 (–2.88 to –2.30)
Higher risk	192	66 (34)	1.71 (0.92 to 3.18)	.09	–2.51 (–2.79 to –2.22)
Highest risk	91	33 (36)	4. 48 (2.29 to 8.76)	<.001	–2.46 (–2.80 to –2.11)
* P*-value for trend				<.001	
Full model with race					
Low risk	91	14 (15)	Reference		–3.35 (–3.48 to –3.22)
Intermediate risk	192	36 (19)	0.98 (0.53 to 1.83)	.96	–2.51 (–2.79 to –2.23)
Higher risk	192	59 (31)	1.65 (0.92 to 2.96)	.10	–2.47 (–2.76 to –2.19)
Highest risk	91	38 (42)	3.20 (1.72 to 5.96)	<.001	–2.44 (–2.76 to –2.11)
* P*-value for trend				<.001	

HR: hazard ratio; CI: confidence interval.

## DISCUSSION

In this study, we externally validated the IIgAN-PT in 677 patients from Peking University First Hospital receiving novel therapies through four approaches: (i) At-biopsy model validation in the new drug cohort; (ii) At-biopsy validation restricted to ERA/SGLT2i-only patients (*n* = 82) to isolate disease-modifying drug effects; (iii) Post-biopsy model validation using 1-year follow-up data; and (iv) Post-biopsy model revalidation incorporating novel drugs (ERAs/SGLT2i as RASBs; Nefecon/hydroxychloroquine/telitacicept as immunosuppressants), given a median novel therapy initiation time of 1.4 years post-biopsy. Both models showed poor performance in model fit and calibration in the new drug cohort.

Previous external validations of the IIgAN-PT have mainly focused on the adult At-biopsy model [11–15], with limited studies on the pediatric At-biopsy model and few on the Post-biopsy model [28, 29]. In large-scale external validations from China and Korea (>1000 patients) [11, 12, 14, 15], the full model with race performed well, with C-statistics of 0.81–0.86, calibration slopes of 0.89–1.26 and R^2^_D_ values of 27%–45.2%—all superior to our results (C-statistic: 0.740; calibration slope: 0.48; R^2^_D_: 17.6%). Compared with both the original cohorts and subsequent large-scale external validations, the model performed worse in our new drug cohort.

We analyzed the reasons for the model's poor performance in our new drug cohort. Firstly, our external validation cohort consisted of patients treated with novel therapies, most of whom received more than one new drug. This discrepancy likely reflected the efficacy of modern treatments, as randomized trials have demonstrated. Secondly, although RASB/immunosuppressant usage aligned with reported cohorts, our novel drug cohort had more severe baseline profiles: younger age, lower eGFR, higher MAP, higher proteinuria and greater prevalence of M1/E1/T1-2/crescent lesions.

We made many attempts at the input of medication variables. The 2024 KDIGO guidelines draft have reclassified the treatment into two categories: manage the IgAN-specific drivers of nephron loss and reduce glomerular hyperfiltration and the impact of proteinuria on the tubulointerstitium. As novel treatments, mechanistically, Nefecon targets the terminal ileum, where mucosal IgA synthesis is concentrated within Peyer's patches [30, 31], while telitacicept targets BAFF and APRIL [20, 32], and hydroxychloroquine affects Toll-like receptors and lysosomes [33, 34]. These drugs have high target specificity, especially telitacicept. Traditional immunosuppressants (e.g. corticosteroids) broadly suppress immune activity through non-specific mechanisms. A phase 3 trial showed Sparsentan significantly reduced proteinuria compared with angiotensin II receptor blockers alone [21, 35]. Novel disease-modifying treatments differ mechanistically from traditional immunosuppressants, and new supportive therapies may also differ from RASB, indicating that drug variables in the model may need further clarification.

The IIgAN-PT was developed using traditional Cox regression [7]. While machine learning (ML) approaches—including random forests, support vector machines and XGBoost—have been explored for IgAN prognosis, their performance has not consistently surpassed conventional methods [36–41]. Park *et al*. found that ML-based models predicted effectively but did not outperform the IIgAN-PT [42]. Furthermore, a meta-analysis evaluated the performance of various IgAN prognostic prediction models revealed the performance ranking: COX regression > new ML models > logistic regression [43]. Despite the rise of ML-based models, the IIgAN-PT continues to demonstrate its robustness and superiority, though further refinement is needed, particularly for new drug cohorts.

Our study provides the following key insights for clinical practice. The applicability of IIgAN-PT should be clearly defined, as it may be suitable for risk prediction in patients undergoing traditional treatments but could overestimate risks in those on new drugs [external validation in not receiving novel therapies patients from original cohort (Supplementary data, Table S7) outperformed that in the new-drug cohort]. Additionally, IIgAN-PT has certain limitations, as risk prediction was based on baseline data and data from the following 1–2 years. It can only predict the risk of major outcomes within 80 months, which is a relatively short time. Given the chronic nature of IgAN, accurate long-term prognostic prediction remains critical. Incorporating dynamic data that reflects changes in exposure over time may be an important direction for future models.

This study has several strengths: First, we performed external validation of IIgAN-PT in a new drug cohort, which significantly differs from previous external validations. Second, our new drug cohort includes a large sample size, with complete follow-up data, especially medication information. However, there are some limitations. Firstly, the small sample size (*n* = 82) in the ERA/SGLT2i group may limit the reliability of the validation results for this subgroup. Secondly, we excluded patients without MEST scores, potentially missing high-risk patients with fewer than eight glomeruli on biopsy. Thirdly, in general, our cohort consisted of patients with more severe disease condition than the overall IgAN population, as milder cases are less likely to be regularly followed up in large tertiary medical centers. Lastly, our cohort only included Chinese patients, further external validation is needed in other ethnic groups using the new drug.

In conclusion, we externally validated the At-biopsy and Post-biopsy models of IIgAN-PT using a new drug cohort. Both models showed poor performance in model fit and calibration, requiring further validation in other new drug cohorts. While IIgAN-PT may need refinement to adapt to diverse novel treatment strategies, its solid past performance and high clinical utility suggest significant potential for improvement. Moreover, the results indicated novel therapies can improve prognosis from another perspective.

## Supplementary Material

sfaf251_Supplemental_File

## Data Availability

The data used in this study cannot be shared publicly due to ethical considerations. Please contact the corresponding authors for research needs.
