# Supplementary material for: Novel therapies improve prognosis of IgAN and limit the applicability of the International IgA Nephropathy Prediction Tool
Source: Clin Kidney J. 2025 Aug 7;18(9):sfaf251. doi: 10.1093/ckj/sfaf251 (PMC12445651; doi:10.1093/ckj/sfaf251)
Supplement: sfaf251_Supplemental_File [file sfaf251_supplemental_file.docx]

**Supplemental Tables**

**Supplemental Table 1.** Calculation formulas for full model with and without race of both At-biopsy and Post-biopsy model

**Supplemental Table 2.** Sample size requirements for external validation calculated using the pmsampsize R package

**Supplemental Table 3.** Detailed medication regimens of our external validation cohort patients

**Supplemental Table 4.** Characteristics of participants in the reported cohorts and the external validation cohort at biopsy and at one year post kidney biopsy

**Supplemental Table 5.** Demographic features in subgroups based on percentile of the linear predictor of the full model without race of At-biopsy model

**Supplemental Table 6.** Demographic features in subgroups based on percentile of the linear predictor of the full model with race of At-biopsy model

**Supplemental Table 7.** Discrimination, calibration and model fit measures in external validation cohort of non-users of the new drugs

**Supplemental Figures**

**Supplemental Figure 1.** Model revalidation confirmed the overestimation pattern for the full model with and without race

**Supplemental Figure 2.** Plots according to risk groups showed the differences between 5-year observed risk and predicted risk for the full model with and without race

**Supplemental Figure 3.** Kaplan–Meier curves of the primary outcome between the risk groups showed poor distinguishing ability in intermediate risk, higher risk and highest risk groups in revalidation of models

**Supplemental Table Appendix**

**Supplemental Table Appendix.** TRIPOD Checklist for External Validation of Prognostic Prediction Models

**Supplemental Table 1. Calculation formulas for full model with and without race of both At-biopsy and Post-biopsy model**

| **Prediction Model** | **Model version** | **Formula for predicted risk of the primary renal outcome** |
| --- | --- | --- |
| At-biopsy model  (IIgAN-PT 2019 version) | Full model with race | Predicted risk (time t) = 1 ‐ S_0_(t)^Exp[LP]^  If t <=36 months, then LP = ‐0.351*[sqrt(eGFR)‐8.8] ‐ 0.0002*(MAP‐97) ‐ 0.093*[log(proteinuria) ‐ 0.09] + 0.006*[(MAP* log(proteinuria)) ‐ 8.73] + 0.155*M1 ‐ 0.131*E1 + 0.097*S1 + 0.607*T1 + 1.189*T2 + 0.109*T1*log(proteinuria) ‐ 0.339*T2*log(proteinuria) ‐ 0.016*(age‐38) ‐ 0.396*Chinese_race + 0.408*Japanese_race ‐ 0.431*Other_race + 0.246*RASB ‐ 0.225*immunosuppression  If t > 36 months, then LP = ‐0.351*[sqrt(eGFR)‐8.8] ‐ 0.0002*(MAP‐97) ‐ 0.093*[log(proteinuria) ‐ 0.09] + 0.006*[(MAP* log(proteinuria)) ‐ 8.73] + 0.155*M1 ‐ 0.131*E1 + 0.097*S1 + 0.607*T1 + 1.189*T2 + 0.109*T1*log(proteinuria) ‐ 0.339*T2*log(proteinuria) ‐ 0.016*(age‐38) + 0.818*Chinese_race + 0.408*Japanese_race ‐ 0.431*Other_race + 0.246*RASB ‐ 0.225*immunosuppression  S_0_(t) = 0.9964303 + 0.04392517*[(t+0.1)/100]^0.5^ ‐ 0.1257002*[(t+0.1)/100]   S_0_(60 months) = 0.95494 |
| At-biopsy model  (IIgAN-PT 2019 version) | Full model without race | Predicted risk (time t) = 1 ‐ S_0_(t)^Exp[LP]^  LP = ‐0.320*[sqrt(eGFR)‐8.8] + 0.002*(MAP‐97) ‐ 0.035*[log(proteinuria) ‐ 0.09] + 0.004*[(MAP* log(proteinuria)) ‐ 8.73] + 0.201*M1 ‐ 0.035*E1 + 0.084*S1 + 0.700*T1 + 1.237*T2 + 0.101*T1*log(proteinuria) ‐ 0.321*T2*log(proteinuria) ‐ 0.017*(age‐38) + 0.118*RASB + 0.166*RASB* log(proteinuria) ‐ 0.26*immunosuppression  S_0_(t) = 1.0003754 ‐ 0.1131641*[(t+0.1)/100]^2^ + 0.0964763*[(t+0.1)/100]^2^*log[(t+0.1)/100]  S_0_(60 months) = 0.94176 |
| Post-biopsy model  (IIgAN-PT 2022 version) | Full model with race | Predicted risk (time t) = 1 - S_0_(t)^Exp[LP]^  LP = -0.6505*[sqrt(eGFR)-8.8] + 0.0021*(MAP-97) + 0.2516*[log(proteinuria) - 0.09] + 0.0043*[(MAP* log(proteinuria)) - 8.73] + 0.2230*M1 + 0.0924*E1 + 0.0547*S1 + 0.1886*T1 + 0.2074*T2 - 0.1650*T1*log(proteinuria) - 0.2781*T2*log(proteinuria) -0.0255*(age-38) + 0.4458*Chinese_race + 1.0798*Japanese_race -0.3425*Other_race + 0.2337*RASB - 0.2879*immunosuppression  S_0_(t) = 1.00130-0.00066339*t-0.00362*s1 where s1=max{(t-2.73)/38.41871,0}^3^+[46.94*max{(t-240.86)/38.41871,0}^3^-238.13*max{(t-49.67)/38.41871,0}^3^]/191.19  S_0_(48 months) = 0.96353, S_0_(60 months) = 0.94959 |
| Post-biopsy model  (IIgAN-PT 2022 version) | Full model without race | Predicted risk (time t) = 1 - S_0_(t)^Exp[LP]^  LP = -0.5862*[sqrt(eGFR)-8.8] - 0.0012*(MAP-97) + 0.1225*[log(proteinuria) - 0.09] + 0.0055*[(MAP* log(proteinuria)) - 8.73] + 0.2693*M1 + 0.2447*E1 - 0.0050*S1 + 0.3237*T1 + 0.4221*T2 - 0.1732*T1*log(proteinuria) - 0.3120*T2*log(proteinuria) -0.0227*(age-38) + 0.2675*RASB - 0.0478*RASB* log(proteinuria) -0.3266*immunosuppression  S_0_(t) = 0.9983872-0.0033270*[(t+0.1)/10]0.5-0.0039851*{[(t+0.1)/10]0.5}*log[(t+0.1)/10], for T<12 months S_0_(t) = 1.00651-0.00105*t-0.00281*s1, for T>=12 months where s1=max{(t-2.73)/38.41871,0}^3^+[46.94*max{(t-240.86)/38.41871,0}^3^-238.13*max{(t-49.67)/38.41871,0}^3^]/191.19   S_0_(48 months) = 0.95151, S_0_(60 months) = 0.93427 |

**Supplemental Table 2. Sample size requirements for external validation calculated using the pmsampsize R package**

| Prediction Model | Version | Sample size |
| --- | --- | --- |
| At-biopsy model | Full model with race | 448 |
|  |  |  |
|  | Full model without race | 469 |
|  |  |  |
| Post-biopsy model | Full model with race | 213 |
|  |  |  |
|  | Full model without race | 215 |
|  |  |  |

**Supplemental Table 3. Detailed medication regimens of our external validation cohort patients**

| **Number of new drugs** | **Drug Regimen** | **Number of patients** |
| --- | --- | --- |
| 1 | ERAs | 10 |
| 1 | Nefecon | 0 |
| 1 | SGLT2i | 38 |
| 1 | Hydroxychloroquine | 255 |
| 1 | Telitacicept | 0 |
| 2 | ERAs+Nefecon | 2 |
| 2 | ERAs+SGLT2i | 34 |
| 2 | ERAs+Hydroxychloroquine | 69 |
| 2 | ERAs+Telitacicept | 2 |
| 2 | Nefecon+SGLT2i | 0 |
| 2 | Nefecon+Hydroxychloroquine | 6 |
| 2 | Nefecon+Telitacicept | 0 |
| 2 | SGLT2i+Hydroxychloroquine | 102 |
| 2 | SGLT2i+Telitacicept | 0 |
| 2 | Hydroxychloroquine+Telitacicept | 2 |
| 3 | ERAs+Nefecon+SGLT2i | 1 |
| 3 | ERAs+Nefecon+Hydroxychloroquine | 1 |
| 3 | ERAs+Nefecon+Telitacicept | 0 |
| 3 | ERAs+SGLT2i+Hydroxychloroquine | 126 |
| 3 | ERAs+SGLT2i+Telitacicept | 2 |
| 3 | ERAs+Hydroxychloroquine+Telitacicept | 0 |
| 3 | Nefecon+SGLT2i+Hydroxychloroquine | 4 |
| 3 | Nefecon+SGLT2i+Telitacicept | 4 |
| 3 | Nefecon+Hydroxychloroquine+Telitacicept | 0 |
| 3 | SGLT2i+Hydroxychloroquine+Telitacicept | 4 |
| 4 | ERAs+Nefecon+SGLT2i+Hydroxychloroquine | 9 |
| 4 | ERAs+Nefecon+SGLT2i+Telitacicept | 0 |
| 4 | ERAs+Nefecon+Hydroxychloroquine+Telitacicept | 1 |
| 4 | ERAs+SGLT2i+Hydroxychloroquine+Telitacicept | 5 |
| 4 | Nefecon+SGLT2i+Hydroxychloroquine+Telitacicept | 0 |
| 5 | ERAs+Nefecon+SGLT2i+Hydroxychloroquine+Telitacicept | 0 |

**Supplemental Table 4.** **Characteristics of participants in the reported cohorts and the external validation cohort at biopsy and at one year post kidney biopsy**

| **Characteristics** | **Reported derivation cohort** | **Reported validation cohort** | **Our validation cohort** |
| --- | --- | --- | --- |
| Number of patients | 2507 | 722 | 566 |
| Follow-up time, median (IQR), yr | 3.9 (2.1, 6.5) | 4.5 (2.4, 7.0) | 4.4 (2.0, 7.9) |
| Year of biopsy, median (IQR) | 2005 (2003, 2008) | 2004 (1999, 2006) | 2017 (2014, 2021) |
| Age, median (IQR), yr | 36 (29, 46) | 36 (29, 46) | 35 (30, 43) |
| Male, n (%) | 1474 (58.8) | 398 (55.1) | 261 (46) |
| Race, n (%) |  |  |  |
| Caucasian | 1112 (44.4) | 187 (25.9) | 0 (0) |
| Chinese | 390 (15.6) | 197 (27.3) | 566 (100) |
| Japanese | 983 (39.2) | 288 (39.9) | 0 (0) |
| Other | 22 (0.9) | 49 (6.8) | 0 (0) |
| eGFR at biopsy, median (IQR),  mL/min per 1.73 m^2^ | 83 (57, 108) | 80 (60, 103) | 75 (51, 97) |
| eGFR at 1 yr, median (IQR),  mL/min per 1.73 m^2^ | 84 (58, 108) | 78 (60, 101) | 67 (49, 83) |
| MAP at biopsy, median (IQR), mmHg | 96.7 (89.3, 106.7) | 94.5 (85.8, 103.3) | 100 (91, 110) |
| MAP at 1 yr, median (IQR), mmHg | 93.3 (86.7, 101.7) | 91.2 (83.3, 100.0) | 103 (95, 110) |
| Proteinuria at biopsy, median (IQR), g/d | 1.2 (0.7, 2.2) | 1.3 (0.8, 2.3) | 1.35 (0.81, 2.50) |
| Proteinuria at 1 yr, median (IQR), g/d | 0.5 (0.2, 1.0) | 0.7 (0.3, 1.5) | 0.74 (0.46, 1.27) |
| MEST histologic score, n (%) |  |  |  |
| M1 | 941 (37.5) | 476 (65.9) | 296 (52) |
| E1 | 399 (15.9) | 303 (42) | 214 (38) |
| S1 | 1925 (76.8) | 546 (75.6) | 427 (75) |
| T1 | 589 (23.5) | 142 (19.7) | 154 (27) |
| T2 | 101 (4) | 46 (6.4) | 52 (9.2) |
| Crescents | 809 (32.3) | 377 (52.2) | 381/556 (68.5) ^a^ |
| Medication use for RASB at biopsy, n (%) | 781 (31.2) | 337 (46.7) | 228 (40) |
| Medication use for RASB at 1 yr, n (%) | 2008 (80.1) | 510 (70.6) | 516 (91) |
| Immunosuppressant use before biopsy, n (%) | 214 (8.5) | 109 (15.1) | 53 (9.4) |
| Immunosuppressant use before 1 yr, n (%) | 804 (32.1) | 245 (33.9) | 336 (59) |
| Primary outcome, n (%) |  |  |  |
| 50% decline in eGFR | 306 (12.2) | 112 (15.5) | 144 (25) |
| ESKD | 236 (9.4) | 88 (12.2) | 110 (19) |
| Total primary outcomes | 385 (15.4) | 123 (17) | 147 (26) |

Data are presented median (Q1, Q3), mean ± standard deviation or *n* (%).

a: Data are shown as n/n (%) because of incomplete crescent data.

**Supplemental Table 5. Demographic features in subgroups based on percentile of the linear predictor of the full model without race of At-biopsy model**

| **Characteristics** | Low risk | Intermediate risk | Higher risk | Highest risk |
| --- | --- | --- | --- | --- |
| Number of patients | 109 | 230 | 229 | 109 |
| Age, median (IQR), yr | 33 (28-38) | 33 (29-41) | 35 (29-42) | 33 (29-42) |
| Male, n (%) | 42 (39) | 99 (43) | 111 (48) | 61 (56) |
| Scr at biopsy, median (IQR), μmol/L | 68 (60-81) | 85 (74-98) | 116 (96-144) | 178 (141-216) |
| eGFR at biopsy, median (IQR), mL/min per 1.73 m^2^ | 111 (98-119) | 88 (76-101) | 61 (48-77) | 36 (30-48) |
| MAP at biopsy, median (IQR), mmHg | 93 (85-102) | 97 (90-106) | 100 (91-108) | 107 (99-119) |
| Proteinuria at biopsy, median (IQR), g/d | 0.56 (0.38-0.88) | 1.23 (0.83-1.78) | 1.79 (1.15-2.95) | 3.36 (2.05-4.54) |
| MEST histologic score, n (%) |  |  |  |  |
| M1 | 24 (22) | 92 (40) | 139 (61) | 89 (82) |
| E1 | 28 (26) | 84 (37) | 94 (41) | 44 (40) |
| S1 | 60 (55) | 161 (70) | 194 (85) | 99 (91) |
| T1 | 2 (2) | 13 (6) | 102 (45) | 62 (57) |
| T2 | 0 (0) | 0 (0) | 15 (7) | 44 (40) |
| RASB use before or at biopsy, n (%) | 31 (28) | 78 (34) | 81 (35) | 38 (35) |
| Immunosuppressant use before or at biopsy, n (%) | 10 (9) | 14 (6) | 19 (8) | 10 (9) |
| Total primary outcomes, n (%) | 28 (26) | 52 (23) | 72 (31) | 38 (35) |

**Supplemental Table 6. Demographic features in subgroups based on percentile of the linear predictor of the full model with race of At-biopsy model**

| **Characteristics** | Low risk | Intermediate risk | Higher risk | Highest risk |
| --- | --- | --- | --- | --- |
| Number of patients | 109 | 230 | 229 | 109 |
| Age, median (IQR), yr | 33 (28-37) | 33 (29-41) | 34 (29-42) | 34 (29-43) |
| Male, n (%) | 40 (37) | 102 (44) | 109 (48) | 62 (57) |
| Scr at biopsy, median (IQR), μmol/L | 68 (60-81) | 85 (74-98) | 115 (95-142) | 178 (142-215) |
| eGFR at biopsy, median (IQR), mL/min per 1.73 m^2^ | 111 (101-119) | 88 (76-101) | 62 (49-77) | 37 (30-46) |
| MAP at biopsy, median (IQR), mmHg | 93 (85-103) | 97 (89-106) | 100 (91-108) | 107 (99-119) |
| Proteinuria at biopsy, median (IQR), g/d | 0.6 (0.4-0.9) | 1.2 (0.8-1.8) | 1.8 (1.2-2.9) | 3.5 (2.3-4.9) |
| MEST histologic score, n (%) |  |  |  |  |
| M1 | 26 (24) | 93 (40) | 138 (60) | 87 (80) |
| E1 | 32 (29) | 85 (37) | 90 (39) | 43 (39) |
| S1 | 61 (56) | 160 (70) | 195 (85) | 98 (90) |
| T1 | 3 (3) | 17 (7) | 101 (44) | 58 (53) |
| T2 | 0 (0) | 0 (0) | 16 (7) | 43 (39) |
| RASB use before or at biopsy, n (%) | 24 (22) | 79 (34) | 87 (38) | 38 (35) |
| Immunosuppressant use before or at biopsy, n (%) | 8 (7) | 15 (7) | 19 (8) | 11 (10) |
| Total primary outcomes, n (%) | 26 (24) | 55 (24) | 68 (30) | 41 (38) |

**Supplemental Table 7.** **Discrimination, calibration and model fit measures in external validation cohort of non-users of the new drugs**

| **Prediction Tool** | **At-biopsy model** | |
| --- | --- | --- |
| Participants | Patients not receiving novel therapies (N=1257^a^) | |
| Variable | With Race | Without Race |
| C statistic | 0.789 | 0.791 |
| Calibration slope | 0.66 | 0.72 |
| ICI | 0.15 | 0.12 |
| R²_D_, % | 38.8 | 42.1 |

ICI: Integrated Calibration Index.

a: Note: Among the 1,331 non-users of the new drugs, 1,257 had complete data for all model variables and were included in the external validation cohort.


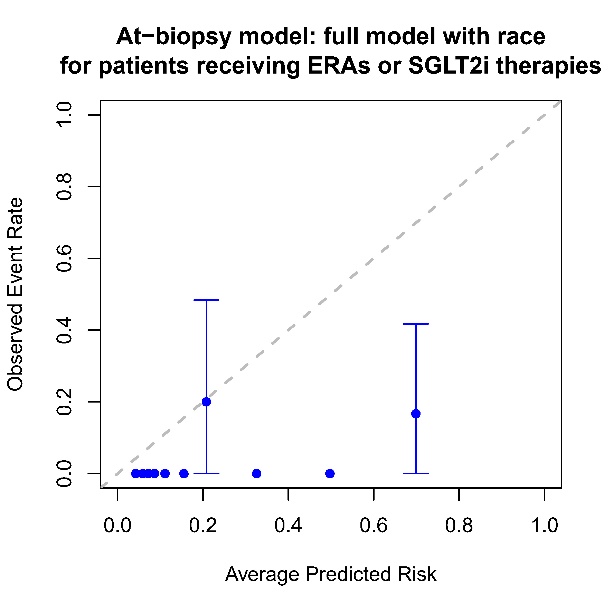

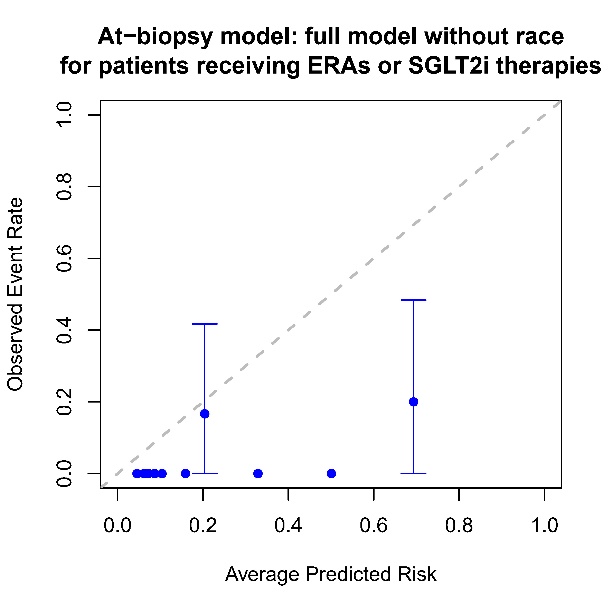

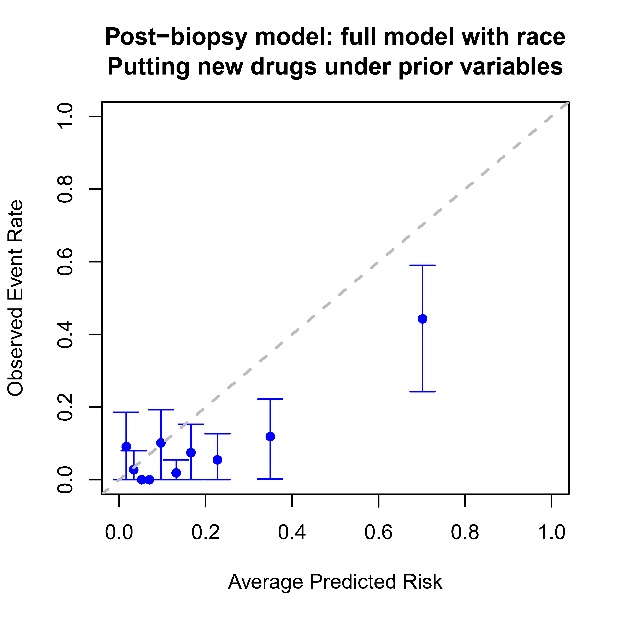

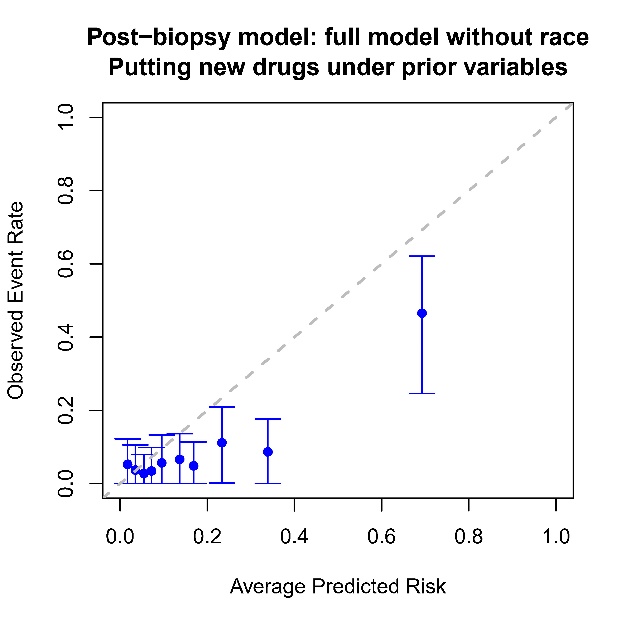


**a**

**c**

**b**

**d**

**Supplemental Figure 1. Model revalidation confirmed the overestimation pattern for the full model with and without race** (a-b) the At-biopsy model was retested in patients receiving ERAs or SGLT2i therapies, (c-d) the Post-biopsy model was retested in putting new drugs under prior variables. Patients were divided into 10 groups according to the deciles of predicted risk derived from each version of each model. The 5-year observed risk and predicted risk at were compared among groups. The dashed line represents perfect calibration, namely the predicted risk is exactly the same as the observed risk. Vertical lines parallel to the vertical axis represent the 95%CI of the observed risk for each group.


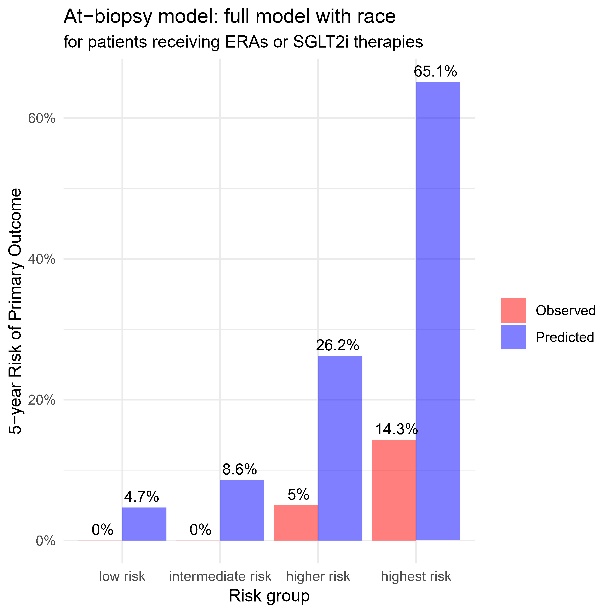

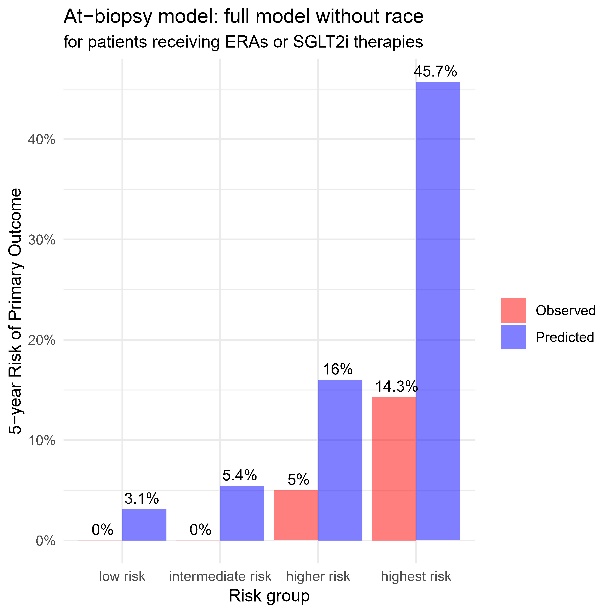

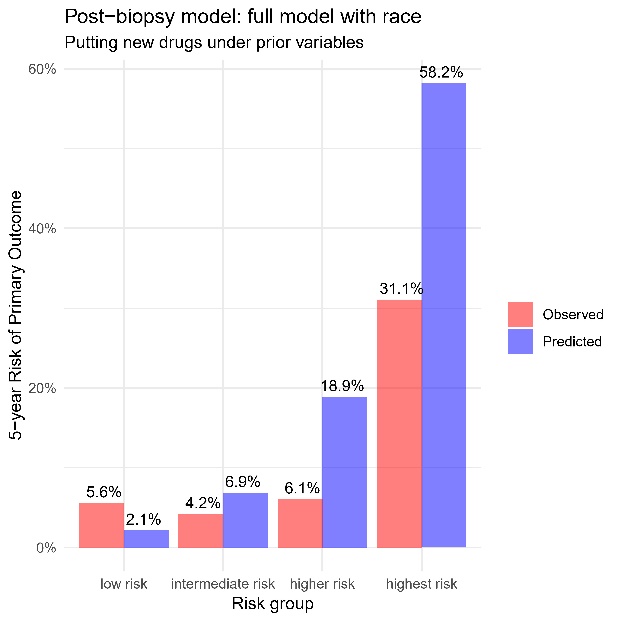

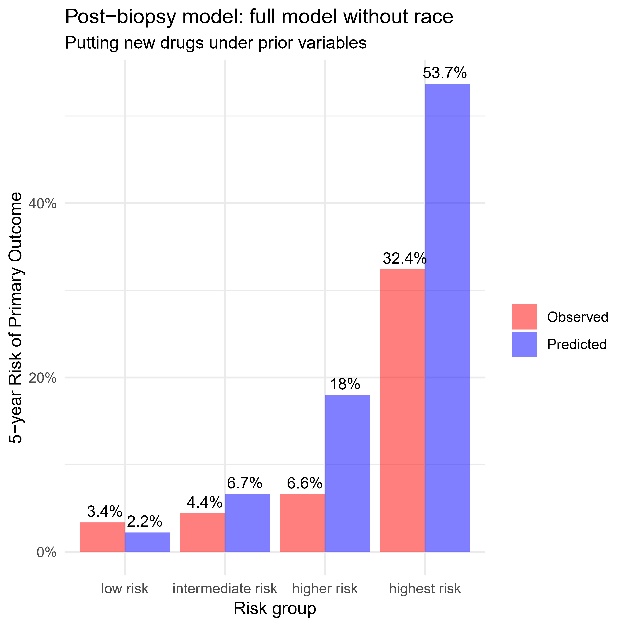


**a**

**c**

**b**

**d**

**Supplemental Figure 2.** **Plots according to risk groups showed the differences between 5-year observed risk and predicted risk for the full model with and without race** (a-b) the At-biopsy model was retested in patients receiving ERAs or SGLT2i therapies, (c-d) the Post-biopsy model was retested in putting new drugs under prior variables. Risk groups were on the basis of percentiles of the linear predictor (low risk: <16th; intermediate risk: 16th to 50th; higher risk: 50th to 84th; and highest risk: >84th).


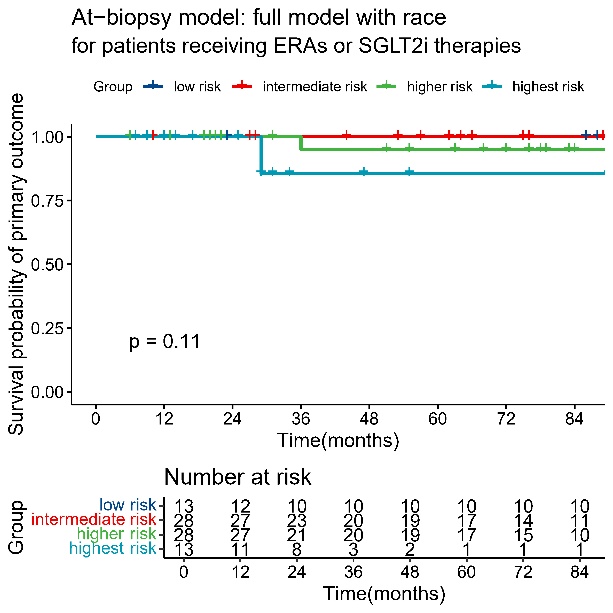

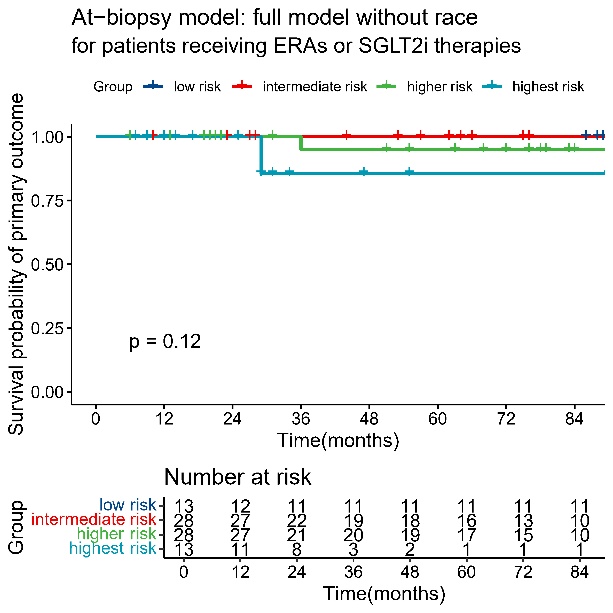

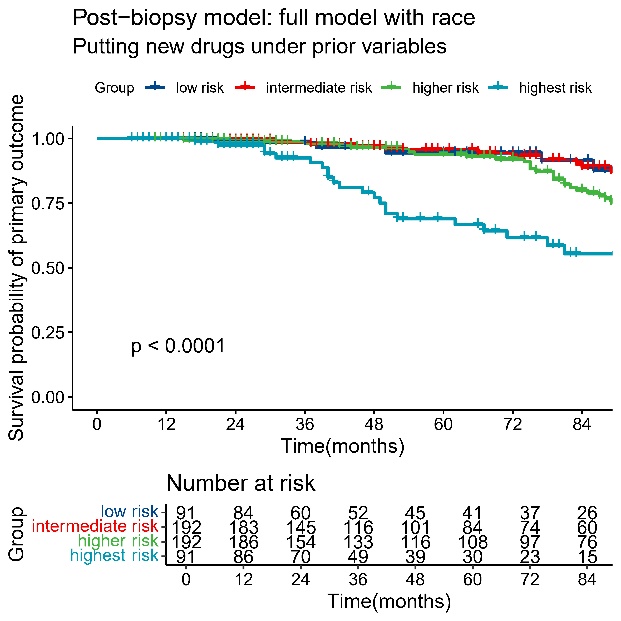

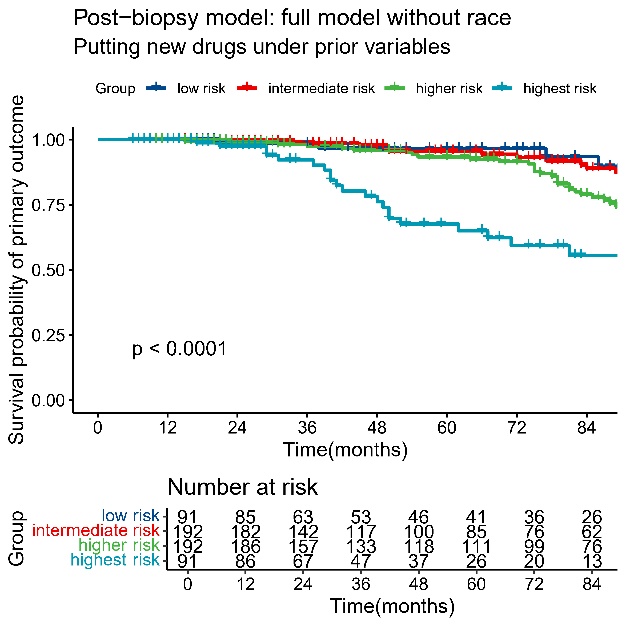


**a**

**c**

**b**

**d**

**Supplemental Figure 3. Kaplan–Meier curves of the primary outcome between the risk groups showed poor distinguishing ability in intermediate risk, higher risk and highest risk groups in revalidation of models** (a-b) the At-biopsy model was retested in patients receiving ERAs or SGLT2i therapies, (c-d) the Post-biopsy model was retested in putting new drugs under prior variables. Risk groups were on the basis of percentiles of the linear predictor (low risk: <16th; intermediate risk: 16th to 50th; higher risk: 50th to 84th; and highest risk: >84th).

**Supplemental Table Appendix. TRIPOD Checklist for External Validation of Prognostic Prediction Models**

|  |  | Reporting Item | Page Number |
| --- | --- | --- | --- |
| **Title** |  |  |  |
|  | [#1](https://www.goodreports.org/reporting-checklists/tripod/info/#1) | Identify the study as developing and / or validating a multivariable prediction model, the target population, and the outcome to be predicted. | 1 |
| **Abstract** |  |  |  |
|  | [#2](https://www.goodreports.org/reporting-checklists/tripod/info/#2) | Provide a summary of objectives, study design, setting, participants, sample size, predictors, outcome, statistical analysis, results, and conclusions. | 3 |
| **Introduction** |  |  |  |
|  | [#3a](https://www.goodreports.org/reporting-checklists/tripod/info/#3a) | Explain the medical context (including whether diagnostic or prognostic) and rationale for developing or validating the multivariable prediction model, including references to existing models. | 5-6 |
|  | [#3b](https://www.goodreports.org/reporting-checklists/tripod/info/#3b) | Specify the objectives, including whether the study describes the development or validation of the model or both. | 5-6 |
| **Methods** |  |  |  |
| Source of data | [#4a](https://www.goodreports.org/reporting-checklists/tripod/info/#4a) | Describe the study design or source of data (e.g., randomized trial, cohort, or registry data), separately for the development and validation data sets, if applicable. | 6 |
| Source of data | [#4b](https://www.goodreports.org/reporting-checklists/tripod/info/#4b) | Specify the key study dates, including start of accrual; end of accrual; and, if applicable, end of follow-up. | 6 |
| Participants | [#5a](https://www.goodreports.org/reporting-checklists/tripod/info/#5a) | Specify key elements of the study setting (e.g., primary care, secondary care, general population) including number and location of centres. | 6 |
| Participants | [#5b](https://www.goodreports.org/reporting-checklists/tripod/info/#5b) | Describe eligibility criteria for participants. | 6 |
| Participants | [#5c](https://www.goodreports.org/reporting-checklists/tripod/info/#5c) | Give details of treatments received, if relevant | supplemental materials page 5 |
| Outcome | [#6a](https://www.goodreports.org/reporting-checklists/tripod/info/#6a) | Clearly define the outcome that is predicted by the prediction model, including how and when assessed. | 6-7 |
| Outcome | [#6b](https://www.goodreports.org/reporting-checklists/tripod/info/#6b) | Report any actions to blind assessment of the outcome to be predicted. | NA^a^ |
| Predictors | [#7a](https://www.goodreports.org/reporting-checklists/tripod/info/#7a) | Clearly define all predictors used in developing or validating the multivariable prediction model, including how and when they were measured | 6 |
| Predictors | [#7b](https://www.goodreports.org/reporting-checklists/tripod/info/#7b) | Report any actions to blind assessment of predictors for the outcome and other predictors. | NA^a^ |
| Sample size | [#8](https://www.goodreports.org/reporting-checklists/tripod/info/#8) | Explain how the study size was arrived at. | 7 |
| Missing data | [#9](https://www.goodreports.org/reporting-checklists/tripod/info/#9) | Describe how missing data were handled (e.g., complete-case analysis, single imputation, multiple imputation) with details of any imputation method. | 6 |
| Statistical analysis methods | [#10a](https://www.goodreports.org/reporting-checklists/tripod/info/#10a) | If you are developing a prediction model describe how predictors were handled in the analyses. | Not developing |
| Statistical analysis methods | [#10b](https://www.goodreports.org/reporting-checklists/tripod/info/#10b) | If you are developing a prediction model, specify type of model, all model-building procedures (including any predictor selection), and method for internal validation. | Not developing |
| Statistical analysis methods | [#10c](https://www.goodreports.org/reporting-checklists/tripod/info/#10c) | If you are validating a prediction model, describe how the predictions were calculated. | 7-8 |
| Statistical analysis methods | [#10d](https://www.goodreports.org/reporting-checklists/tripod/info/#10d) | Specify all measures used to assess model performance and, if relevant, to compare multiple models. | 7-8 |
| Statistical analysis methods | [#10e](https://www.goodreports.org/reporting-checklists/tripod/info/#10e) | If you are validating a prediction model, describe any model updating (e.g., recalibration) arising from the validation, if done | No updating |
| Risk groups | [#11](https://www.goodreports.org/reporting-checklists/tripod/info/#11) | Provide details on how risk groups were created, if done. | 7 |
| Development vs. validation | [#12](https://www.goodreports.org/reporting-checklists/tripod/info/#12) | For validation, identify any differences from the development data in setting, eligibility criteria, outcome, and predictors. | 6-7 |
| **Results** |  |  |  |
| Participants | [#13a](https://www.goodreports.org/reporting-checklists/tripod/info/#13a) | Describe the flow of participants through the study, including the number of participants with and without the outcome and, if applicable, a summary of the follow-up time. A diagram may be helpful. | 8 |
| Participants | [#13b](https://www.goodreports.org/reporting-checklists/tripod/info/#13b) | Describe the characteristics of the participants (basic demographics, clinical features, available predictors), including the number of participants with missing data for predictors and outcome. | 8 |
| Participants | [#13c](https://www.goodreports.org/reporting-checklists/tripod/info/#13c) | For validation, show a comparison with the development data of the distribution of important variables (demographics, predictors and outcome). | 8 |
| Model development | [#14a](https://www.goodreports.org/reporting-checklists/tripod/info/#14a) | If developing a model, specify the number of participants and outcome events in each analysis. | Not developing |
| Model development | [#14b](https://www.goodreports.org/reporting-checklists/tripod/info/#14b) | If developing a model, report the unadjusted association, if calculated between each candidate predictor and outcome. | Not developing |
| Model specification | [#15a](https://www.goodreports.org/reporting-checklists/tripod/info/#15a) | If developing a model, present the full prediction model to allow predictions for individuals (i.e., all regression coefficients, and model intercept or baseline survival at a given time point). | Not developing |
| Model specification | [#15b](https://www.goodreports.org/reporting-checklists/tripod/info/#15b) | If developing a prediction model, explain how to the use it. | Not developing |
| Model performance | [#16](https://www.goodreports.org/reporting-checklists/tripod/info/#16) | Report performance measures (with CIs) for the prediction model. | 9 |
| Model-updating | [#17](https://www.goodreports.org/reporting-checklists/tripod/info/#17) | If validating a model, report the results from any model updating, if done (i.e., model specification, model performance). | No updating |
| **Discussion** |  |  |  |
| Limitations | [#18](https://www.goodreports.org/reporting-checklists/tripod/info/#18) | Discuss any limitations of the study (such as nonrepresentative sample, few events per predictor, missing data). | 11 |
| Interpretation | [#19a](https://www.goodreports.org/reporting-checklists/tripod/info/#19a) | For validation, discuss the results with reference to performance in the development data, and any other validation data | 10 |
| Interpretation | [#19b](https://www.goodreports.org/reporting-checklists/tripod/info/#19b) | Give an overall interpretation of the results, considering objectives, limitations, results from similar studies, and other relevant evidence. | 10-11 |
| Implications | [#20](https://www.goodreports.org/reporting-checklists/tripod/info/#20) | Discuss the potential clinical use of the model and implications for future research | 11 |
| **Other information** |  |  |  |
| Supplementary information | [#21](https://www.goodreports.org/reporting-checklists/tripod/info/#21) | Provide information about the availability of supplementary resources, such as study protocol, Web calculator, and data sets. | Supplemental materials |
| Funding | [#22](https://www.goodreports.org/reporting-checklists/tripod/info/#22) | Give the source of funding and the role of the funders for the present study. | 12 |

Note: None The TRIPOD checklist is distributed under the terms of the Creative Commons Attribution License CC-BY. This checklist can be completed online using <https://www.goodreports.org/>, a tool made by the [EQUATOR Network](https://www.equator-network.org) in collaboration with [Penelope.ai](https://www.penelope.ai)

a: Since the data were derived from automated electronic records, blinding of outcome assessors was not feasible, though the potential bias was deemed minimal.
